# P-993. Shorter is Better: Evaluating the Impact of a Microlearning Initiative for Antimicrobial Stewardship

**DOI:** 10.1093/ofid/ofaf695.1192

**Published:** 2026-01-11

**Authors:** Steven Doerstling, Ritika Prasad, Emily Mui, Will Alegria, David R Ha, Radhika Arya, Alex Zimmet, Stan Deresinski, Marisa Holubar, Lina Meng

**Affiliations:** Stanford University School of Medicine, Palo Alto, California; Stanford Health Care, Stanford, California; Stanford University School of Medicine, Palo Alto, California; Stanford University School of Medicine, Palo Alto, California; Stanford Health Care, Stanford, California; Stanford University School of Medicine, Palo Alto, California; Stanford Healthcare, Stanford University School of Medicine, Palo Alto, California; Stanford Health Care, Stanford, California; Stanford University School of Medicine, Palo Alto, California; Stanford Health Care, Stanford, California

## Abstract

**Background:**

Microlearning is a strategy to deliver short, focused material and may be useful for busy clinicians. The Stanford Antimicrobial Safety & Sustainability Program developed a microlearning curriculum for antimicrobial stewardship (AS), called “ABX Pearls,” emailed weekly to clinicians and trainees across several service lines at Stanford Health Care. This project aimed to understand the impact of ABX Pearls on clinical practice across specialties and training backgrounds.

**Methods:**

Recipients of the ABX Pearls emails were invited to complete an online survey about their experiences with the microlearning content. The following groups were included: internal medicine (residents, attendings, and advanced practice providers [APPs]), critical care (attendings and APPs), pharmacists, and APPs from other subspecialty services.

**Results:**

The response rate was 20% (103/517). Our sample included 17 residents (17%), 22 attendings (21%), 44 pharmacists (43%), and 20 APPs (19%) (Table 1). Most (89%) respondents indicated reading the ABX Pearls emails more than half the time, and 97% reported that the content being “short and easy to read” was a feature they liked most. 81% said they have used the ABX Pearls to make patient management decisions, including a high proportion of residents (94%) and attendings (95%) but a lower proportion of APPs (63%) (Table 2). ABX Pearls influenced a number of AS skills, and all groups reported that “increased awareness of institutional resources” was the AS skill with the most significant impact (Figure 1). When treating infections, respondents identified distinct challenges by training background; diagnostic stewardship was most challenging for residents (76%) and attendings (76%), antimicrobial resistance for pharmacists (68%), and antibiotic spectrum of activity for APPs (95%) (Figure 2).

**Conclusion:**

An email-based microlearning curriculum for AS was highly engaging and impacted clinical practice across specialties and training backgrounds. Different clinical roles present unique challenges when treating infections, and microlearning is an effective way to address them. Further study is needed to verify the reported change in clinical management and compare to other didactics and methods of distribution.

**Disclosures:**

All Authors: No reported disclosures

